# Comprehensive Evaluation of the Quality of *Tripterygium* Glycosides Tablets Based on Multi-Component Quantification Combined with an In Vitro Biological Assay

**DOI:** 10.3390/molecules27165102

**Published:** 2022-08-10

**Authors:** Yadan Wang, Zhong Dai, Jiangong Yan, Xianfu Wu, Shuangcheng Ma

**Affiliations:** 1 National Institutes for Food and Drug Control, Beijing 102629, China; 2 Traditional Chinese Medicine Processing Technology Innovation Center of Hebei Province, College of Pharmacy, Hebei University of Chinese Medicine, Shijiazhuang 050200, China; 3 State Key Laboratory of Bioactive Substance and Function of Natural Medicines, Institute of Materia Medica, Chinese Academy of Medical Sciences and Peking Union Medical College, Beijing 100050, China

**Keywords:** *Triptergium* glycosides tablets (TGTs), RRLC–ESI–MS/MS, anti-inflammatory activity, cytotoxicity, quality consistency, triptolide, triptoquinone B, celastrol, demethylzelaysteral

## Abstract

*Tripterygium* glycosides tablets (TGTs) are widely used in clinical practice to treat rheumatoid arthritis and other autoimmune diseases, with significant beneficial effects but also high toxicity, necessitating rigorous quality evaluation and control. In current study, a rapid resolution liquid chromatography tandem electrospray ionization triple quadrupole mass spectrometry (RRLC–ESI–MS/MS) method was developed and validated for the quantitative analysis of 14 components of ten batches of TGTs produced by different manufacturers, including four diterpenoids, three triterpenoids, and seven sesquiterpene alkaloids. Meanwhile, the NO inhibition effects of these TGTs were evaluated in LPS-induced RAW264.7 cells for their downstream anti-inflammatory activities, as well as their cytotoxicity. The results indicate that the TGTs from different manufacturers showed poor quality consistency, as evidenced by large variations in chemical profiles and biological effects, which may increase the risks associated with clinical use. To improve the quality status of TGTs, it is crucial to identify indicator components whose characterization can accurately reflect the efficacy and toxicity of TGTs from which they were derived. Our study reveals that triptolide, triptoquinone B, celastrol, and demethylzelaysteral considerably contributed to the anti-inflammatory activity and/or cytotoxicity of TGTs, implying that they should be further investigated as candidate indicator components for TGT quality control.

## 1. Introduction

*Tripterygium wilfordii* Hook. F (TwHF) has been extensively used in traditional Chinese medicine (TCM) for centuries to treat an array of autoimmunological disorders, including rheumatoid arthritis (RA), lupus erythematosus and nephrotic syndrome [1,2]. Recent pharmacological studies have shown that TwHF possesses, among others, anti-inflammatory, immunosuppressive and anti-tumor activities [3,4,5]. Chemical investigations on TwHF have revealed three main types of components that are responsible for these effects: diterpenoids, triterpenoids and sesquiterpene alkaloids [6]. Despite the obvious therapeutic effects, the clinical applications of TwHF are restricted due to its narrow therapeutic window and severe adverse effects on organs such as the liver and kidneys in addition to those comprising the reproductive system [7,8,9].

*Tripterygium* glycosides tablets (TGTs), a preparation of TwHF, were developed in the 1980s. Interestingly, despite the name, the preparation contains almost no glycoside components. TGT have become the first-line therapy for RA patients in China, but they are also troubled by a similar toxicity problem to TwHF [10,11]. At present, TGTs are supplied by many manufacturers. Since the production process of TGTs is quite complex, it mainly consists of the following steps: (1) extracting the peeled root of TwHF with ethanol; (2) partitioning the ethanol extract with water and chloroform; (3) the separation of the chloroform extract using silica gel column chromatography to collect several specific fractions; (4) the mixing of the collected fractions at a certain ratio so as to obtain the *Triptergium* glycoside raw materials; (5) the tableting using *Triptergium* glycoside raw materials and an appropriate amount of excipients so that each tablet contains 10 mg of *Triptergium* glycosides; it is particularly challenging to achieve a consistent product quality of TGTs from different manufacturers, which in turn may influence their therapeutic effect and toxicity. Thus, the quality control of TGTs is crucially important to ensure the effectiveness and safety of this preparation. The current quality standard of TGTs was set by China’s Food and Drug Administration (CFDA), which stipulated triptolide and wilforlide A as indicator components; the content of the former should be not exceed 10 μg per tablet, while the content of the latter should be at least 10 μg per tablet. However, due to the complexity of TGT components, the determination of only the above two factors would not sufficiently reflect the quality of TGTs. Therefore, some analytic methods such as liquid chromatography tandem mass spectrometry (LC–MS) and supercritical fluid chromatography–diode array detector–tandem mass spectrometry (SFC–DAD–MS), have been established for the quantitative analysis of multiple components in TGTs [12,13,14]. However, no studies that consider both the preparation’s chemical profile and its biological effects, as well as the potential relationship between the two, have been conducted.

**Figure 1:**
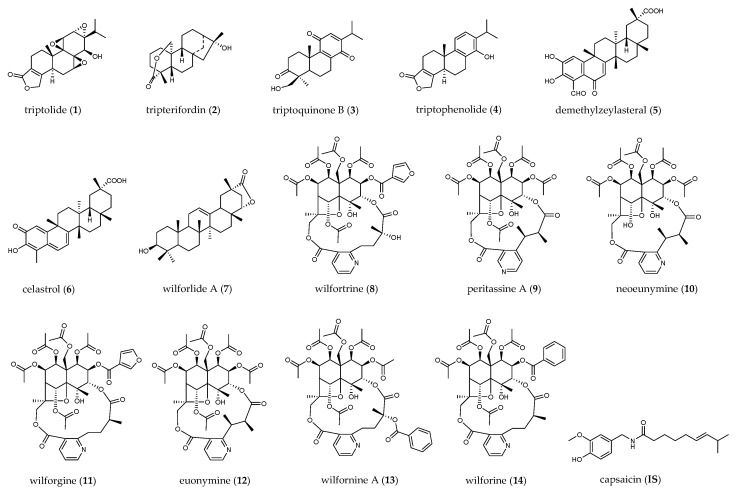
Chemical structures of compounds quantified in TGTs.

In this study, we collected ten batches of TGTs from different manufacturers. Firstly, a rapid resolution liquid chromatography tandem electrospray ionization triple quadrupole mass spectrometry (RRLC–ESI–MS/MS) method was developed for the quantification of 14 bioactive constituents, including four diterpenoids (**1**–**4**), three triterpenoids (**5–7**) and seven sesquiterpene alkaloids (**8**–**14**). Their structures are shown in Figure 1. Capsaicin was used as the internal standard (IS). Since the content levels of sesquiterpene alkaloids are much higher than those of diterpenoids and triterpenoids, they were determined separately using a lower concentration of sample solution. Then, the TGTs were assessed for their inhibitory effects on NO production in LPS-induced RAW264.7 cells as well as their cytotoxicity. Based on the above investigations, the chemical profiles, anti-inflammatory activity, and cytotoxicity of TGTs from different manufacturers were compared, from which we could draw conclusions as to the quality status of this preparation. Furthermore, the biological effects of the 14 target compounds were estimated in order to identify the main ingredients that significantly contribute to the therapeutic activity and/or toxicity of TGTs, which may be used as potential indicator components for the quality control of this preparation.

## 2. Results

### 2.1. Optimization of Extraction Conditions

In order to establish optimal extraction conditions, the variables involved in the whole extraction procedure, including extraction methods (reflux and ultrasonic extraction), extraction solvents (50, 70, and 100% methanol, *v*/*v*), and extraction time (30, 45, and 60 min.) were evaluated regarding their effect on extraction efficiency. The results indicate that the efficiency of ultrasonic extraction is comparable to that of reflux extraction, but has the advantage of being much simpler. It was also found that, of the various concentrations tested, 100% methanol was the most efficient extraction solvent. Furthermore, target compounds could be completely extracted within 45 min. Therefore, the sample solutions were prepared by ultrasonic extraction with 30 mL methanol for 45 min (Data not shown).

### 2.2. Optimization of Chromatographic and Mass Spectrometric Conditions

To achieve the desired chromatographic behavior while maximizing the signal intensity of the analytes and maintaining a short analysis time, the use of various mobile phase systems (methanol–water, acetonitrile–water, methanol–acid aqueous solution, and acetonitrile–acid aqueous solution) was tested. Acetonitrile, with a stronger elution capability, satisfied the requirement of minor peak width and a shorter analysis time. Formic acid was selected as the aqueous solvent additive at a concentration of 0.1% over acetic acid, as it resulted in a satisfactory analyte resolution and peak shape as well as a higher ionization intensity (Figures S1–S4). Therefore, the optimal mobile phase, consisting of acetonitrile and 0.1% formic acid aqueous solution, was finally employed.

**Figure 2:**
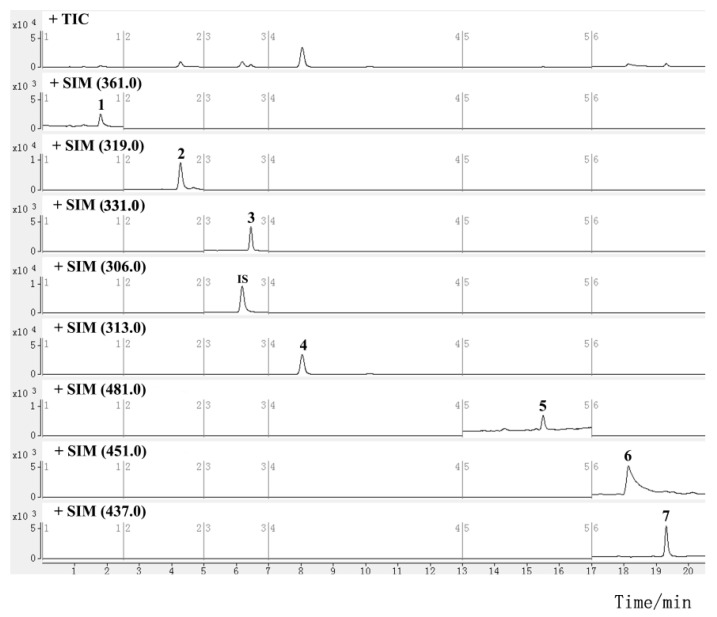
SIM chromatograms for investigated diterpenoids and triterpenoids in TGTs (sample S2, the peak numbers correspond to the compound numbers in Figure 1).

**Figure 3:**
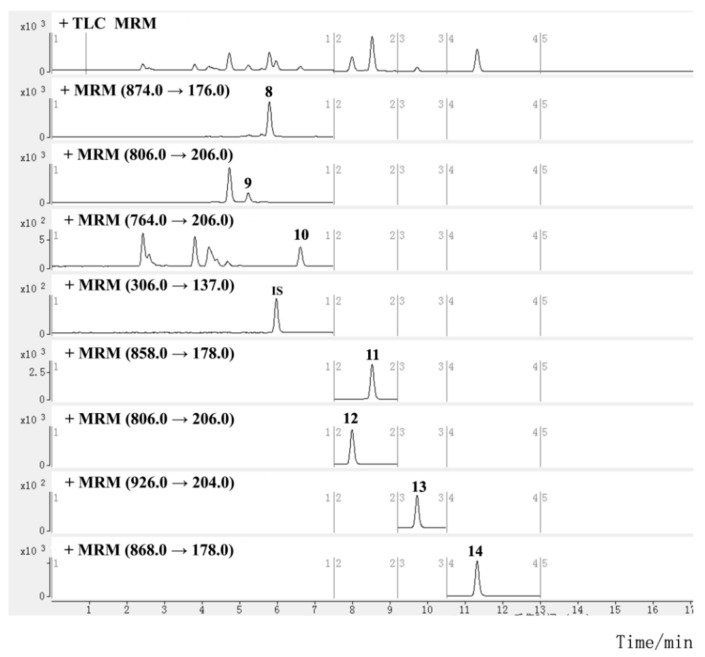
MRM chromatograms for investigated sesquiterpene alkaloids in TGTs (sample S2, the peak numbers correspond to the compound numbers in Figure 1).

Mass spectra were studied in both positive and negative ion modes. All the analytes and IS showed higher ionization efficiencies in the positive mode. For the quantitative analysis, selected ion monitoring (SIM) and multiple reaction monitoring (MRM) methods are commonly used. The former uses a quadrupole for ion selection, and detects the target ion without intentional fragmentation, while the latter uses tandem quadrupoles for ion selection and fragmentation, and detects the precursor/fragment pairs. The MRM method is preferred for compound quantification due to its higher sensitivity and selectivity, but the SIM method is used for compounds that are difficult to fragment or have low or unstable product ion response. Sesquiterpene alkaloids were determined using the MRM method. To obtain the most abundant response of precursor and product ion, the parameters of the fragment voltage (FV) and collision energy (CE) of each alkaloid were optimized. Since the mass fragmentation peaks of some diterpenoids and triterpenoids, such as triptolide, were sensitive to CE and displayed a low response, it was difficult to achieve abundant and stable transitions; they were instead determined using the SIM method with optimized FV (Figures S5 and S6). The retention time (RT) and MS information for diterpenoids and triterpenoids are listed in Table 1, and those for sesquiterpene alkaloids are shown in Table 2. The main fragmentation patterns of the alkaloids used to elucidate the mechanism of selected product ions generation are shown in Figure S7. The typical SIM chromatograms for diterpenoids and triterpenoids are shown in Figure 2, and the MRM chromatograms for sesquiterpene alkaloids are shown in Figure 3.

### 2.3. Method Validation

The method was validated in terms of linearity, limit of quantification (LOQ), limit of detection (LOD), precision, repeatability, stability, and recovery tests. As shown in Table 3 and Table 4, the 14 analytes showed good linear regression with correlation coefficients (R^2^) of more than 0.9990 within their test ranges, and the LOQs and LODs ranged from 0.2 to 7.5 ng/mL and 0.06 to 2.5 ng/mL, respectively. The intra- and inter-day precision of the analytes exhibited an RSD of less than 2.78 and 3.65%, respectively. All the analytes showed good repeatability and stability within 24 h at room temperature (20–25 °C) with RSDs in the range of 0.52–3.93%, and 1.03–3.45%, respectively. The average recoveries of these analytes varied between 97.43 and 103.74%, with RSDs of less than 4.23%, indicating the good reliability and accuracy of the proposed method.

### 2.4. Quantitative Analysis of TGTs from Different Manufacturers

**Figure 4:**
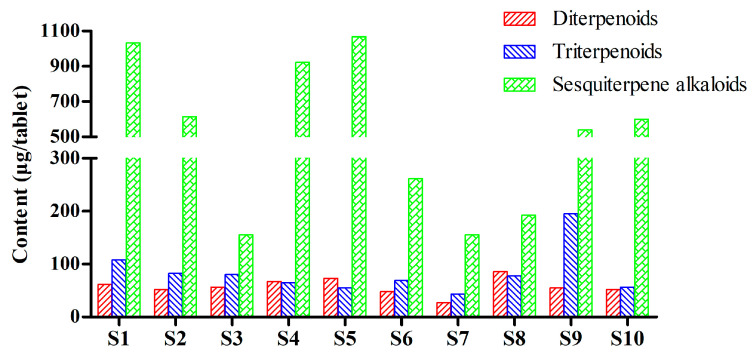
Total contents of the investigated diterpenoids, triterpenoids, and sesquiterpene alkaloids in TGTs from different manufacturers.

The developed RRLC–ESI–MS/MS method was subsequently applied in the quantitative analysis of ten batches of TGTs from different manufacturers. The results are listed in Table 5 and Figure 4. Sesquiterpene alkaloids were present in the preparations in relatively high concentrations compared to the other two types of components, but they also varied the most between manufacturers. The total content ranged from 105.76 to 1067.67 µg/tablet, with above 500 µg/tablet in the samples S1, S2, S4, S5, S9, and S10 and below 300 µg/tablet in the other samples. Among the seven investigated alkaloids, wilfortrine (**8**), paritassine A (**9**), wilforgine (**11**), and wilforine (**14**) were the most abundant in all the samples except for S3, in which wilfortrine was the only alkaloid with a high content. For diterpenoids and triterpenoids, wilforlide A (**7**), an indicator component in the current CFDA quality standard, was relatively abundant in the various TGTs, with contents ranging from 22.15 to 82.82 µg/tablet, with higher levels above 80 g/tablet in S1, moderate levels ranging from 40 to 70 µg/tablet in S2, S3, S4, S5, S6, S8, and S10, and lower levels below 30 µg/tablet in S7 and S9. The contents of the other analytes varied greatly between samples. It is noteworthy that the celastrol (**6**) content in S9 reached 147.88 μg/tablet, which was significantly higher than in other samples. In addition, triptolide (**1**), another standard indicator component, was present in samples at 0–11.67 μg/tablet, with S1, S4 and S5 having higher levels above 9 µg/tablet, S2, S9, and S10 having moderate levels ranging from 5 to 8 µg/tablet, and S3, S6, S7 and S8 having lower levels below 3 µg/tablet, or even having undetectable levels. Tripterifordin (**2**), triptoquinone B (**3**), triptophenolide (**4**), and demethylzeylasteral (**5**) were relatively abundant in S1, S3, S8, and S9, respectively. These results indicate that the chemical profiles of TGTs from different manufacturers are quite different, which may further influence the therapeutic effects and toxicity of the preparations.

### 2.5. Anti-Inflammatory Activities and Cytotoxicities of TGTs

The anti-inflammatory activities of TGTs were evaluated by measuring their inhibitory effects on NO production in LPS-induced RAW 264.7 cells, and their cytotoxicities were assessed using the CCK-8 method. As shown in Table 6 and Figures S8 and S9, TGTs from different manufacturers exhibited widely varying inhibitory effects on NO generation, which can be roughly classified into three groups, with S1–S5, S9, and S10 showing strong effects corresponding to IC_50_ values ranging from 5.41 to 13.06 μg/mL, S7 and S8 showing weaker effects with IC_50_ values of 52.62 and 61.80 μg/mL, respectively, and S6 showing the weakest effect with an IC_50_ value of 189.32 μg/mL. The cytotoxicities of TGTs against RAW264.7 cells, on the other hand, also varied substantially. Interestingly, we discovered that the cytotoxicities of TGTs were correlated with their anti-inflammatory activities, with the more active samples being more toxic and, conversely, the less toxic ones being less active. Furthermore, most TGTs had similar IC_50_ and TC_50_ values, with therapeutic index (TI) values of less than 2.0, confirming the restricted safety window of TGTs in clinical application.

### 2.6. Anti-Inflammatory Activities and Cytotoxicites of the Investigated Compounds

Although there are some reports in the literature of the 14 investigated compounds having anti-inflammatory activity or toxicity [6,15,16,17,18], the relative magnitude of their effects is unclear due to differences in the pharmacological models and assay methods used. In order to elucidate the contribution of each compound to the efficacy and toxicity of TGTs, they were also tested using the same methods as TGTs. As shown in Table 7 and Figures S10 and S11, among all the investigated compounds, triptolide exhibited extremely significant NO inhibition activity and cytotoxicity, with IC_50_ and TC_50_ values of 0.066 and 0.071 μM, respectively. Celastrol and demethylzeylasteral showed slightly weaker anti-inflammatory activities and cytotoxicities with IC_50_ values of 0.56 and 3.48 μM, and TC_50_ values of 1.72 and 25.79 μM, respectively, but the values for their therapeutic indexes were greater than that of triptolide. Triptoquinone B also exhibited potent cytotoxicity against RAW 264.7 cells, with a TC_50_ value of 0.11 μM, comparable to triptolide, but showed only moderate anti-inflammatory activity with an IC_50_ value of 35.65 μM. In addition, triptophenolide showed mild anti-inflammatory activity, with an IC_50_ value of 43.11 μM, but very weak cytotoxicity. The remaining compounds demonstrated negligible NO inhibitory effects and had little influence on cell viability at a concentration of 50 μM. Therefore, it is presumed that, of all the tested compounds, triptolide, celastrol, demethylzeylasteral, and triptoquinone B may have relatively greater influence on the biological effects of TGTs.

Our further analysis further demonstrates that the content variation of these four ingredients could, to some extent, account for the differences in the activity and toxicity of TGTs. First, the six more active/toxic TGTs including S1, S2, S4, S5, S9, and S10 contained more than 5 μg/tablet of triptolide, whereas the three less active TGTs contained relatively low levels of triptolide, particularly S6, which had almost no anti-inflammatory activity and toxicity. When we added 100 μg of triptolide into ten tablets of S6, the anti-inflammatory activity and cytotoxicity were significantly increased, with IC_50_ and TC_50_ values of 6.48 and 10.68 μg/mL, respectively (Figures S12 and S13), further supporting the crucial role of triptolide in the activity and toxicity of TGTs. Second, S9 had a lower triptolide content when compared with S2 and S10 but showed a greater anti-inflammatory activity, probably due to the much higher levels of demethylzeylasteral and celastrol. Third, S3 exhibited potent cytotoxicity, which is partly attributed to the higher level of triptoquinone B. However, the above four compounds were not the only factors determining the biological effects of TGTs; for example, S3 contained low amounts of triptolide, demethylzeylasteral and celastrol but showed the most significant NO inhibition effect, thus indicating that there must be other additional active components in the preparations that merit further investigation.

## 3. Discussion

In this study, the quality of TGTs from different manufacturers was comprehensively evaluated for the first time through multi-component quantification in combination with an in vitro biological assay. As a clinically common and highly efficacious and toxic preparation, the quality status of TGTs is not promising, as indicated by the wide variation in their chemical composition, anti-inflammatory activity, and cytotoxicity, which may increase the risks associated with clinical use. There are two main reasons for the varying quality of TGTs: first, the manufacturing process of TGTs is complicated, particularly regarding the step of silica column chromatography, which makes it challenging for different manufacturers to achieve high quality conformance. Second, the selection of indicator components and statutory contents in the current standard for TGTs quality is not satisfactory, as they cannot be effectively reflected and therefore be used in controlling the quality of TGTs. The standard specifies the upper limit of triptolide, based mainly on the consideration of its toxicity. However, a number of studies, including this one, have confirmed that triptolide is one of the main active components in TwHF, with significant anti-inflammatory, immunosuppressive, and anti-tumor effects, among others [15,19]. As a result, it is considered more reasonable to control a specified content range of triptolide rather than its upper limit. Furthermore, a minimum content limit for another indicator component, wilforlide A, is also specified in the standard. Our study indicates that wilforlide A has negligible anti-inflammatory activity and cytotoxicity on top of its extremely low oral bioavailability reported in the literature [20]. Therefore, wilforlide A dosage not appear to reflect the quality of TGTs and should not be used as an indicator. Instead, celastrol, demethylzeylasteral, and triptoquinone B, which were found to have potent activity and/or toxicity in this study, can be further investigated as candidate indicator components. Their absorption and metabolism parameters were predicted using the SwissADME tool, and it was found that triptolide and triptoquinone B may have good gastrointestinal absorption with a moderate bioavailability of 0.55, while demethylzeylasteral and celastrol may have relatively poor absorption, but celastrol has a high predicted bioavailability of 0.85 (Table S1). The data presented above supported, to some extent, the selection of these candidate index components, but they still require further validation. In addition to the abovementioned ingredients, many other active and/or toxic components that exist in TwHF need to be studied, especially the derivatives of triptolide that have similar biological effects to triptolide, such as tripdiolide, triptonide, and tripchlorolide, among others [21]. Indicator components used in TGT quality control should be selected according to their content, activity, and toxicity, with the minimum limit of content specified for indicators with high activity but low toxicity, the upper limit of content specified for indicators with low activity but high toxicity, and a content range specified for indicators with both high activity and toxicity.

## 4. Materials and Methods

### 4.1. Chemicals and Reagents

Ten batches of TGTs from different manufacturers (S1–S10) were collected from the market. The reference standards of triptolide (**1**), triptophenolide (**4**), celastrol (**6**) and wilforlide A (**7**) and capsaicin (IS) were supplied by National Institutes for Food and Drug Control (NIFDC, Beijing, China). Tripterifordin (**2**), demethylzeylasteral (**5**), wilfortrine (**8**), peritassine A (**9**), wilforgine (**11**), euonymine (**12**), wilfornine A (**13**), and wilforine (**14**) were purchased from Shanghai Standard Technology Co., Ltd. (Shanghai, China). Triptoquinone B (**3**) and neoeunymine (**10**) were isolated from the root of TwHF in our laboratory. The structures of these compounds were identified by comparing the MS, and ^1^H- and ^13^C-NMR spectra with the literature data, and all compounds were determined to have >98% purity based on HPLC–UV analysis.

Acetonitrile (MS grade) and methanol (HPLC grade) were obtained from Fisher Scientific Inc. (FairLawn, OH, USA). Ultrapure water was prepared by a Mili-Q water purification system (Milipore, Burlington, MA, USA). LPS were purchased from Sigma-Aldrich (St. Louis, MO, USA). Other chemicals and reagents were of analytical grade.

### 4.2. Preparation of Standard Solutions

A stock solution containing four diterpenoids and three triterpenoids standards was prepared by dissolving the reference standards in methanol to a final concentration of 3.59 μg/mL for triptolide (**1**), 10.02 μg/mL for tripterifordin (**2**), 15.00 μg/mL for triptoquinone B (**3**), 3.10 μg/mL for triptophenolide (**4**), 40.88 μg/mL for demethylzeylasteral (**5**), 21.96 μg/mL for celastrol (**6**), and 23.50 μg/mL for wilforlide A (**7**). Another stock solution containing seven sesquiterpene alkaloids was prepared as described above with a concentration of 1.72 μg/mL for wilfortrine (**8**), 1.62 μg/mL for peritassine A (**9**), 0.14 μg/mL for neoeunymine (**10**), 3.25 μg/mL for wilforgine (**11**), 0.76 μg/mL for euonymine (**12**), 1.12 μg/mL for wilfornine A (**13**), and 1.02 μg/mL for wilforine (**14**). The two solutions were diluted separately with methanol to obtain a series of working solutions, to which capsaicin (IS) was added to produce final concentrations of 0.11 and 0.055 μg/mL, respectively. All solutions were stored at 4 °C and brought to room temperature before use.

### 4.3. Preparation of Sample Solutions

Twenty pills of each batch of TGTs were accurately weighed to determine the average tablet weight, and were then powdered. A portion of the powder (equivalent to 10 tablets) was accurately weighted and ultrasonically extracted with 30 mL of methanol for 45 min, and then cooled to room temperature. Methanol was added to compensate for the loss of weight, followed by filtering of the solution. For the diterpenoid and triterpenoid analysis, the filtrate was diluted 10 times with methanol, and an appropriate amount of capsaicin (IS) solution was added to obtain a final concentration of 0.11 μg/mL. For the sesquiterpene alkaloid analysis, the filtrate was diluted 100 times with methanol, and the final concentration of IS was 0.055 μg/mL. All the resultant solutions were filtered through a 0.22 μm nylon filter before RRLC–ESI–MS/MS analysis.

### 4.4. RRLC–ESI–MS/MS Conditions

Liquid chromatography was performed on an Agilent Series 1200 system (Agilent Technologies, Santa Clara, CA, USA) equipped with a degasser, binary pump, autosampler and thermostatted column compartment. Chromatographic separation was performed on an Agilent Extend C18 column (2.1 mm × 100 mm, 1.8 μm) at 30 °C. The mobile phase consisted of 0.1% formic acid aqueous solution (A) and acetonitrile (B). A gradient elution of 40–50% B at 0–10 min, 50–80% B at 10–15 min, 80–95% B at 15–20 min, and 95–95% B at 20–25 min was used for the diterpenoid and triterpenoid analysis. An alternative gradient elution of 40–50% B at 0–10 min, 50–68% B at 10–13 min, and 95–95% B at 13–20 min was used for the sesquiterpene alkaloid analysis. The flow rate was set at 0.3 mL/min. The injection volume was 2 μL.

All MS experiments were conducted on a 6410B triple quadrupole mass spectrometer (Agilent Technologies, Palo Alto, CA, USA) equipped with an ESI source. The analytes were determined in positive ionization mode, with the SIM method used for diterpenoids and triterpenoids and the MRM method for sesquiterpene alkaloids. Data acquisition was performed using a Mass Hunter Workstation. Capillary voltage was set to 4000 V. Desolvation gas (nitrogen) was delivered at 540 L/h and 350 °C. Nebulizer pressure was set to 0.2 MPa.

### 4.5. Method Validation

#### 4.5.1. Linearity, LOQs, and LODs

The calibration curves of at least six concentration levels of each standard were constructed from the peak area ratio of the analyte to IS versus their concentrations. The LOQs and LODs were determined at signal-to-noise (S/N) ratios of about 10 and 3, respectively.

#### 4.5.2. Precision, Repeatability, and Stability

The precision of the method was assessed in terms of intra- and inter-day variations. Intermediate concentration standard solutions were analyzed six times within one day for the intra-day test, and in triplicate on three consecutive day for the inter-day test. To confirm the repeatability, six different sample solutions prepared from the same sample (S2) were analyzed. The concentration of each solution was determined according to a calibration curve that was derived on the same day. Variations are expressed as RSD. Stability was tested using one of the sample solutions (S2), which was stored at room temperature (20–25 °C) and analyzed at 0, 2, 4, 8, 12, and 24 h.

#### 4.5.3. Recovery

A recovery test was performed to ensure the accuracy of the established method. Accurate amounts of the 14 standards at low (80% of the known amount), medium (100% of the known amount), and high (120% of the known amount) levels were added to the same sample (S2) in triplicate. Then, the spiked samples were extracted and analyzed using the abovementioned method. The average recoveries were calculated using the following formula: recovery (%) = (detected amount − original amount)/spiked amount × 100.

### 4.6. Cell Culture

The RAW 264.7 macrophage cell line was obtained from ATCC (Manassas, VA, USA). Cells were cultured at 37 °C in a humidified air incubator with 5% CO_2_ in Dulbecco’s modified Eagle’s medium (DMEM; Gibco, Grand Island, NY, USA) supplemented with 10% fetal bovine serum (FBS; Gibco, USA), 100 units/mL penicillin and 100 µg/mL streptomycin (Gibco, USA).

### 4.7. Anti-Inflammation Assay

RAW 264.7 cells (5.0 × 10^4^ cells/well) were seeded into a 96-well plate and incubated overnight. The culture medium was replaced with DMEM medium containing various concentrations of TGT or target compound, subsequently treated with LPS (1 µg/mL), and then incubated for 24 h. After 50 µL of cultured medium was transferred to a new 96-well plate, 50 µL of Griess reagent was added to each well. The absorbance at 540 nm was determined using a microplate reader (PerkinElmer, Waltham, MA, USA). The concentration of NO in the culture supernatants was calculated by using a standard curve of nitrite, a major stable product of NO, which was generated according to the application instructions for the Griess reagent.

### 4.8. Cytotoxicity Assay

RAW 264.7 cells (5.0 × 10^4^ cells/well) were seeded into a 96-well plate. After overnight incubation, various concentrations of TGT or target compound were applied to the cells, which were then incubated for 24 h. The same volume of DMSO was added to the compound-untreated group to account for any effects of DMSO on cell viability. Then, the culture medium was removed, 100 μL DMEM medium plus 10 μL CCK-8 reagent (MCE, Suzhou, China) was added, and the cells were incubated for another 2 h at 37 °C in a 5% CO_2_ incubator. Absorbance at 450 nm was read using a microplate reader, and cell viability was calculated as the percentage of absorbance of various concentrations versus the control group.

## Figures and Tables

**Table 1:** Retention time and related MS parameters for investigated diterpenoids, triterpenoids, and IS.

Analytes	Retention Time (min)	Selected Ion (*m/z)*	Fragment Voltage (V)
triptolide (**1**)	1.80	361.0 [M + H]^+^	150
tripterifordin (**2**)	4.27	319.0 [M + H]^+^	50
triptoquinone B (**3**)	6.45	331.0 [M + H]^+^	200
triptophenolide (**4**)	8.04	313.0 [M + H]^+^	125
demethylzeylasteral (**5**)	15.50	481.0 [M + H]^+^	150
celastrol (**6**)	18.14	451.0 [M + H]^+^	125
wilforlide A (**7**)	19.31	437.0 [M − H_2_O + H]^+^	175
capsaicin (IS)	6.18	306.0 [M + H]^+^	75

**Table 2:** Retention time and related MS parameters for investigated sesquiterpene alkaloids and IS.

Analytes	Retention Time (min)	Precursor Ion (*m/z*)	Product Ion (*m/z*)	Fragment Voltage (V)	Collision Energy (eV)
wilfortrine (**8**)	5.79	874.0 [M + H]^+^	176.0	175	40
peritassine A (**9**)	5.66	806.0 [M + H]^+^	206.0	175	40
neoeunymine (**10**)	6.62	764.0 [M + H]^+^	206.0	175	40
wilforgine (**11**)	8.53	858.0 [M + H]^+^	178.0	175	60
euonymine (**12**)	7.69	806.0 [M + H]^+^	206.0	175	40
wilfornine A (**13**)	9.72	926.0 [M + H]^+^	204.0	175	50
Wilforine (**14**)	11.32	868.0 [M + H]^+^	178.0	175	70
capsaicin (IS)	5.97	306.0 [M + H]^+^	137.0	75	30

**Table 3:** Calibration curves, limit of detection (LOD), limit of quantification (LOQ), precision, repeatability, and stability for the 14 analytes.

Analytes	Calibration Curves	R^2^	Linear Range (µg/mL)	LOQ (ng/mL)	LOD (ng/mL)	Precision (RSD, %)		Repeatability (RSD, %, *n* = 6)	Stability (RSD, %, *n* = 6)
						Intra-Day (*n* = 6)	Inter-Day (*n* = 3)		
triptolide	*Y* = 0.7269 *X* − 0.0054	1.0000	0.00090–3.59	0.9	0.3	0.73	1.78	2.07	2.14
tripterifordin	*Y* = 0.0837 *X* + 0.0019	0.9997	0.00050–1.00	0.5	0.2	1.09	2.03	2.43	2.65
triptoquinone B	*Y* = 0.0453 *X* − 0.0111	0.9990	0.0075–15.00	7.5	2.5	2.78	3.65	3.93	3.07
triptophenolide	*Y* = 6.1908 *X* − 0.0469	0.9991	0.0016–1.55	1.6	0.5	1.54	3.12	2.71	2.98
demethylzeylasteral	*Y* = 0.2556 *X* + 0.0031	0.9995	0.0020–8.18	2.0	0.6	1.85	1.74	3.64	1.87
celastrol	*Y* = 1.9742 *X* + 0.0063	0.9999	0.00022–4.39	0.2	0.06	0.99	2.05	0.52	1.55
wilforlide A	*Y* = 0.0517 *X* − 0.0003	0.9995	0.0059–2.35	5.9	2.0	2.25	2.66	1.66	3.45
wilfortrine	*Y* = 4.4918 *X* + 0.0110	0.9998	0.00086–1.72	0.9	0.3	0.34	1.54	1.71	1.23
peritassine A	*Y* = 1.6421 *X* − 0.0001	0.9997	0.0013–1.62	1.3	0.5	1.06	1.23	1.79	1.55
neoeunymine	*Y* = 21.280 *X* − 0.0010	0.9997	0.00023–0.14	0.2	0.08	1.14	2.23	1.66	2.47
wilforgine	*Y* = 7.0238 *X* − 0.0839	0.9993	0.0011–1.08	1.1	0.4	0.35	0.89	1.39	1.90
euonymine	*Y* = 13.977 *X* + 0.0001	0.9998	0.00063–0.76	0.6	0.2	0.86	1.12	1.51	2.55
wilfornine A	*Y* = 2.6602 *X* − 0.0007	0.9997	0.0019–1.12	1.9	0.6	0.82	2.32	1.72	1.03
wilforine	*Y* = 4.7038 *X* + 0.0527	0.9995	0.0010–1.02	1.0	0.3	0.85	1.67	1.01	1.24

**Table 4:** Recoveries of the 14 analytes.

Analytes	Original (μg)	Spiked (μg)	Detected (μg)	Mean Recovery (%) (RSD, %, n = 3)
triptolide	32.78	23.08	55.92	100.26 (2.01)
		38.48	70.79	98.78 (1.30)
		53.86	88.39	103.24 (1.92)
tripterifordin	58.78	37.88	97.09	101.14 (1.11)
		63.13	120.86	98.34 (1.94)
		88.38	148.10	101.06 (2.55)
triptoquinone B	118.04	90.00	207.09	98.94 (2.48)
		150.00	268.25	100.14 (2.26)
		210.00	324.40	98.27 (2.03)
triptophenolide	50.08	27.92	78.50	101.80 (3.39)
		46.53	96.31	99.36 (1.59)
		65.14	114.62	99.08 (2.28)
demethylzeylasteral	31.50	27.59	59.72	102.26 (3.83)
		45.99	77.17	99.30 (3.20)
		64.39	98.21	103.60 (1.55)
celastrol	154.16	86.47	242.99	102.73 (2.71)
		144.11	298.70	100.30 (4.23)
		201.75	361.67	102.85 (1.88)
wilforlide A	299.45	144.67	373.47	99.56 (2.86)
		241.11	472.41	100.77 (2.84)
		337.55	570.56	101.05 (3.45)
wilfortrine	596.35	388.08	983.21	99.69 (2.45)
		646.80	1250.69	101.17 (2.81)
		905.52	1525.84	102.65 (1.40)
peritassine A	381.92	243.45	623.69	99.31 (1.69)
		405.75	802.83	103.74 (1.47)
		568.05	960.18	101.80 (1.65)
neoeunymine	26.90	20.41	46.91	98.04 (2.50)
		34.02	60.53	98.87 (2.62)
		47.63	74.62	100.20 (2.60)
wilforgine	792.10	487.80	1270.60	98.09 (0.96)
		813.00	1600.98	99.49 (0.62)
		1138.20	1911.79	98.37 (0.86)
euonymine	175.92	113.85	290.24	100.41 (1.53)
		189.75	364.51	99.39 (0.73)
		265.65	443.02	100.55 (1.81)
wilfornine A	272.20	168.53	438.62	98.75 (3.79)
		280.88	545.86	97.43 (1.50)
		393.23	659.75	98.56 (2.31)
wilforine	824.66	457.20	1279.68	99.52 (2.09)
		762.00	1584.73	99.75 (1.51)
		1066.80	1870.15	98.00 (0.80)

**Table 5:** Quantitative results for the 14 analytes in TGTs from different manufacturers.

No	Content (μg/Tablet)													
	1 ^a^	2	3	4	5	6	7	8	9	10	11	12	13	14
S1	11.67	36.06	13.12	0.66	5.71	19.61	82.82	151.38	153.00	10.36	307.64	107.42	91.38	211.27
S2	6.57	11.80	23.68	10.05	6.32	30.29	46.03	119.63	76.61	5.40	158.89	35.29	54.60	165.43
S3	2.58	0.079	53.66	0.20	11.32	20.94	48.26	150.08	3.16	tr ^b^	1.42	tr	0.61	0.49
S4	10.81	25.06	15.09	16.50	0.92	18.35	45.75	158.86	150.95	8.06	270.70	88.49	86.48	159.08
S5	9.95	14.13	18.64	30.58	0.46	5.46	49.30	284.37	230.42	6.38	260.08	97.59	54.59	134.24
S6	tr	23.26	8.743	16.09	nd ^c^	2.14	66.94	46.46	25.90	2.33	101.22	21.55	12.40	51.96
S7	1.59	7.96	12.68	5.12	2.66	18.51	22.15	20.94	36.48	1.13	59.37	11.04	2.86	23.95
S8	1.22	20.90	21.43	42.45	nd	10.25	67.48	29.81	41.09	1.59	63.31	12.20	6.20	38.43
S9	5.75	13.21	20.07	16.16	21.42	147.88	26.10	128.04	76.51	5.80	161.96	39.29	18.90	109.38
S10	7.95	18.99	18.64	6.45	1.04	15.03	40.59	87.70	91.95	7.64	186.88	55.46	48.77	122.68

^a^ The numbers correspond to the compound numbers in Figure 1; ^b^ trace, lower than LOQ; ^c^ not detected, lower than LOD.

**Table 6:** NO inhibitory effects of TGTs in LPS-induced RAW 264.7 cells and their cytotoxicities against RAW 264.7 cells.

No.	NO Inhibitory Effect IC_50_ (μg/mL ^a^)	Cytotoxicity TC_50_ (μg/mL ^a^)	Therapeutic Index (TI, = TC_50_/IC_50_)
S1	7.72	10.12	1.31
S2	9.91	10.58	1.07
S3	5.41	8.78	1.62
S4	13.06	19.20	1.47
S5	11.01	16.32	1.48
S6	189.32	nu ^b^	– ^c^
S7	52.62	75.03	1.43
S8	61.80	75.93	1.23
S9	6.12	9.12	1.49
S10	6.26	10.55	1.69

^a^ The units of IC_50_ and TC_50_ values are in terms of the concentrations of *Tripterygium glycosides* raw materials. ^b^ Had no influence on cell viability at a concentration of 300 μg/mL; ^c^ cannot be calculated.

**Table 7:** NO inhibitory effects of investigated compounds in LPS-induced RAW 264.7 cells and their cytotoxicities against RAW 264.7.

No.	NO Inhibitory Effect IC_50_ (μM)	Cytotoxicity TC_50_ (μM)	Therapeutic Index (TI, = TC_50_/IC_50_)
triptolide	0.066	0.071	1.08
tripterifordin	nu ^a^	w ^b^	– ^c^
triptoquinone B	35.65	0.11	0.0031
triptophenolide	43.11	w	–
demethylzeylasteral	3.48	27.59	7.93
celastrol	0.56	1.72	3.07
wilforlide A	nu	nu	–
wilfortrine	nu	nu	–
peritassine A	nu	nu	–
neoeunymine	nu	nu	–
wilforgine	w	w	–
euonymine	nu	nu	–
wilfornine A	w	w	–
wilforine	w	w	–

^a^ Had no NO inhibitory effect or cytotoxicity with an NO production rate less than 0 or cell viability more than 100% at a concentration of 50 μM; ^b^ had weak NO inhibitory effect or cytotoxicity with an NO production rate less than 50% or the cell viability more than 50% at a concentration of 50 μM; ^c^ cannot be calculated.

## Data Availability

The data presented in this study are available in article and Supplementary Material.

## Supplementary Materials

The following supporting information can be downloaded at: www.mdpi.com/article/10.3390/molecules27165102/s1, Figures S1–S4: Representative chromatograms obtained using different mobile phase; Figures S5–S6: The fragment profiles of triptolide with different collision energy and in different injections; Figure S7: The main fragmentation patterns of the alkaloids; Figures S8–S13: The NO inhibition and cytotoxicity curves of TGTs and investigated compounds; Table S1: Predicted absorption and metabolism parameters for investigated compounds by SwissADME tool.

## Sample Availability

Not available.
