# Supplementary material for: Comprehensive Evaluation of the Quality of Tripterygium Glycosides Tablets Based on Multi-Component Quantification Combined with an In Vitro Biological Assay
Source: Molecules. 2022 Aug 10;27(16):5102. doi: 10.3390/molecules27165102 (PMC9416487; doi:10.3390/molecules27165102)
Supplement: Supplementary file 1 [file molecules-27-05102-s001.zip › molecules-1826382-supplementary.pdf]

# **Comprehensive evaluation of the quality of *Tripterygium* glycosides tablets based on multi-component quantification combined with in vitro biological assay**

**Yadan Wang [1], Zhong Dai [1], Jiangong Yan [2], Xianfu Wu [1,3,*], and Shuangcheng Ma [1,*]**

[1] National Institutes for Food and Drug Control, Beijing 102629, China
[2] Traditional Chinese Medicine Processing Technology Innovation Center of Hebei Province, Hebei University of Chinese Medicine, Shijiazhuang 050200, China
[3] State Key Laboratory of Bioactive Substance and Function of Natural Medicines, Institute of Materia Medica, Chinese Academy of Medical Science and Peking Union Medical College, Beijing 100050, China

* Correspondence: wuxf99@163.com (X.W.); masc@nifdc.org.cn (S.M.); Tel.: +86-10-5385-2026 (X.W.); +86-10-5385-2076 (S.M.)

## Supplementary data

Figure S1: Representative SIM chromatograms for investigated diterpenoids and triterpenoids in TGTs (S2) using acetonitrile-water as mobile phase........................................................................................................3

Figure S2: Representative SIM chromatograms for investigated diterpenoids and triterpenoids in TGTs (S2) using acetonitrile-0.1% formic acid aqueous solution as mobile phase...........................................................4

Figure S3: Representative MRM chromatograms for investigated alkanoids in TGTs (S2) using acetonitrile-water as mobile phase........................................................................................................5

Figure S4: Representative MRM chromatograms for investigated alkanoids in TGTs (S2) using acetonitrile--0.1% formic acid aqueous solution as mobile phase......................................................................6

Figure S5: The fragment profiles of triptolide with different collision energy.................................................7

Figure S6: The fragment profiles of triptolide with 35 eV of collision energy in two injections.......................7

Figure S7: The main fragmentation patterns of the alkaloids............................................................................8

Figure S8: The NO inhibition curves of 10 batches of *Tripterygium* glycosides tablets (TGTs, S1–S10)..............................................................................................................................................9

Figure S9: The cytotoxicity curves against RAW 264.7 cells of 10 batches of TGTs (S1–S10).......................10

Figure S10: The NO inhibition curves of 14 target compounds........................................................................11

Figure S11: The cytotoxicity curves against RAW 264.7 cells of 14 target compounds.................................12

Figure S12: The NO inhibition curve of S6 added with triptolide (10 μg/tablet) ............................................13

Figure S13: The cytotoxicity curve against RAW 264.7 cells of S6 added with triptolide (10 μg/tablet).......................................................................................................................................13

Table S1: Predicted absorption and metabolism parameters for investigated compounds by SwissADME tool.............................................................................................................................................14

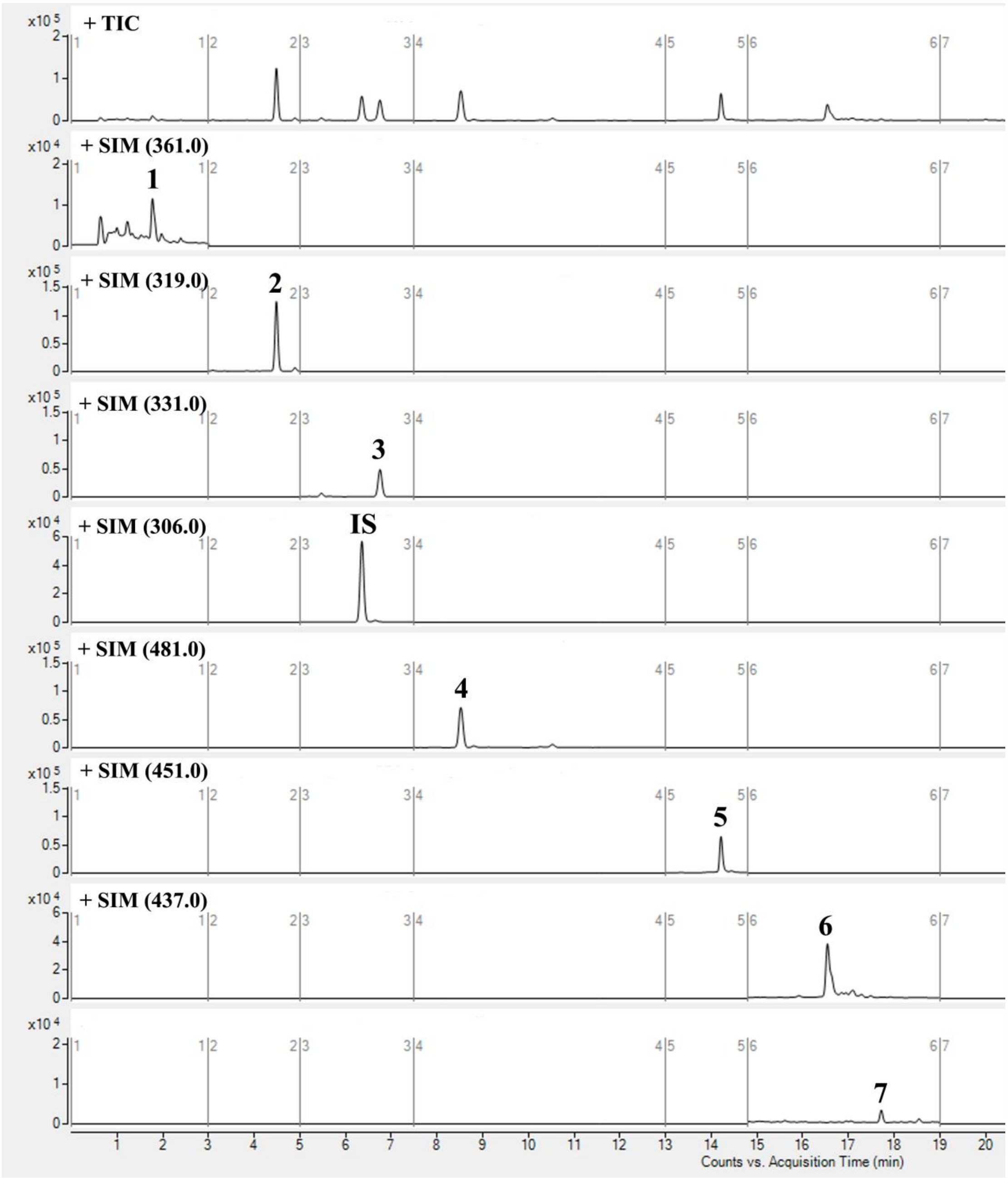


**Figure S1. Representative SIM chromatograms for investigated diterpenoids and triterpenoids in TGTs (S2) using acetonitrile-water as mobile phase**

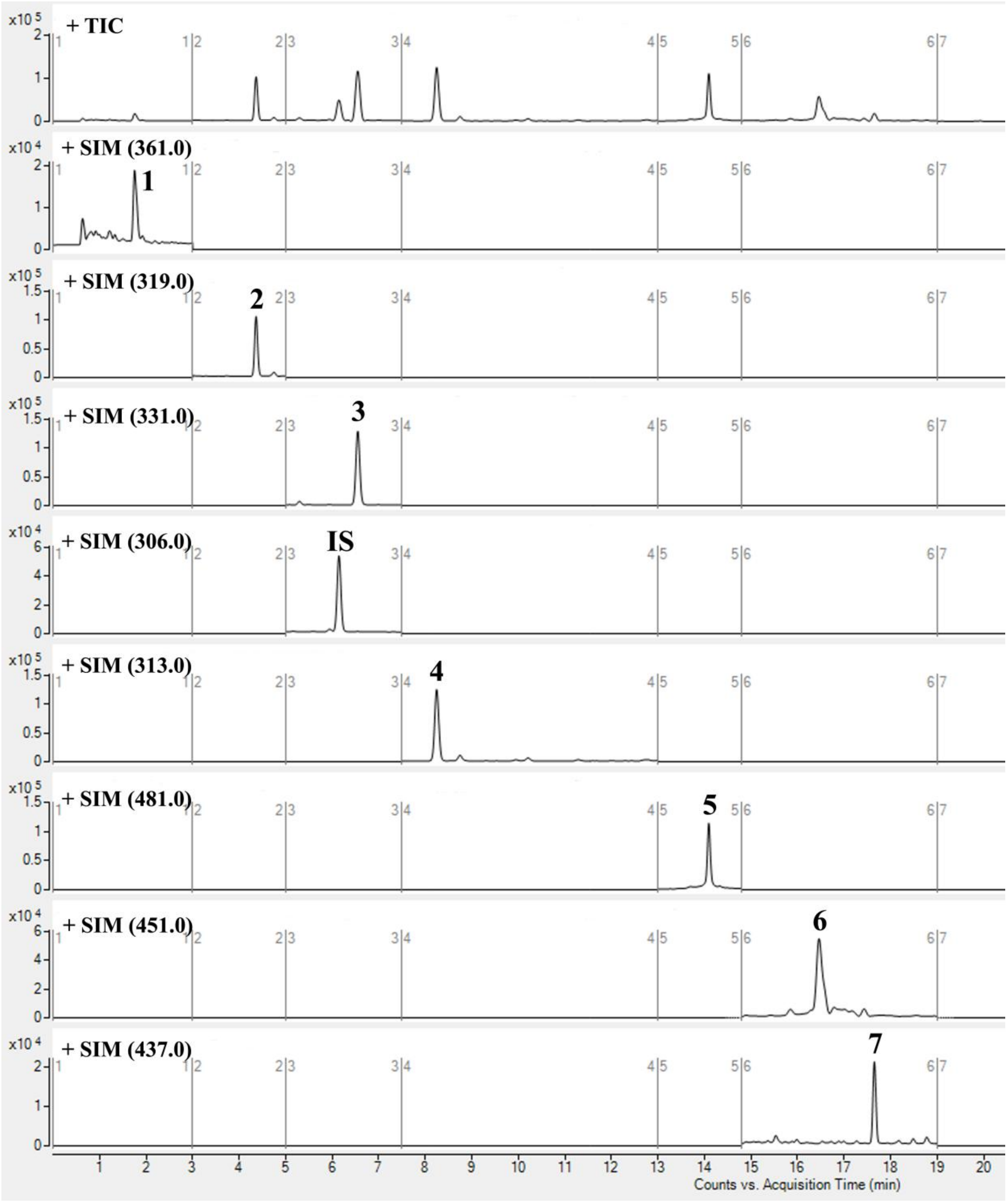


**Figure S2. Representative SIM chromatograms for investigated diterpenoids and triterpenoids in TGTs (S2) using acetonitrile-0.1% formic acid aqueous solution as mobile phase**

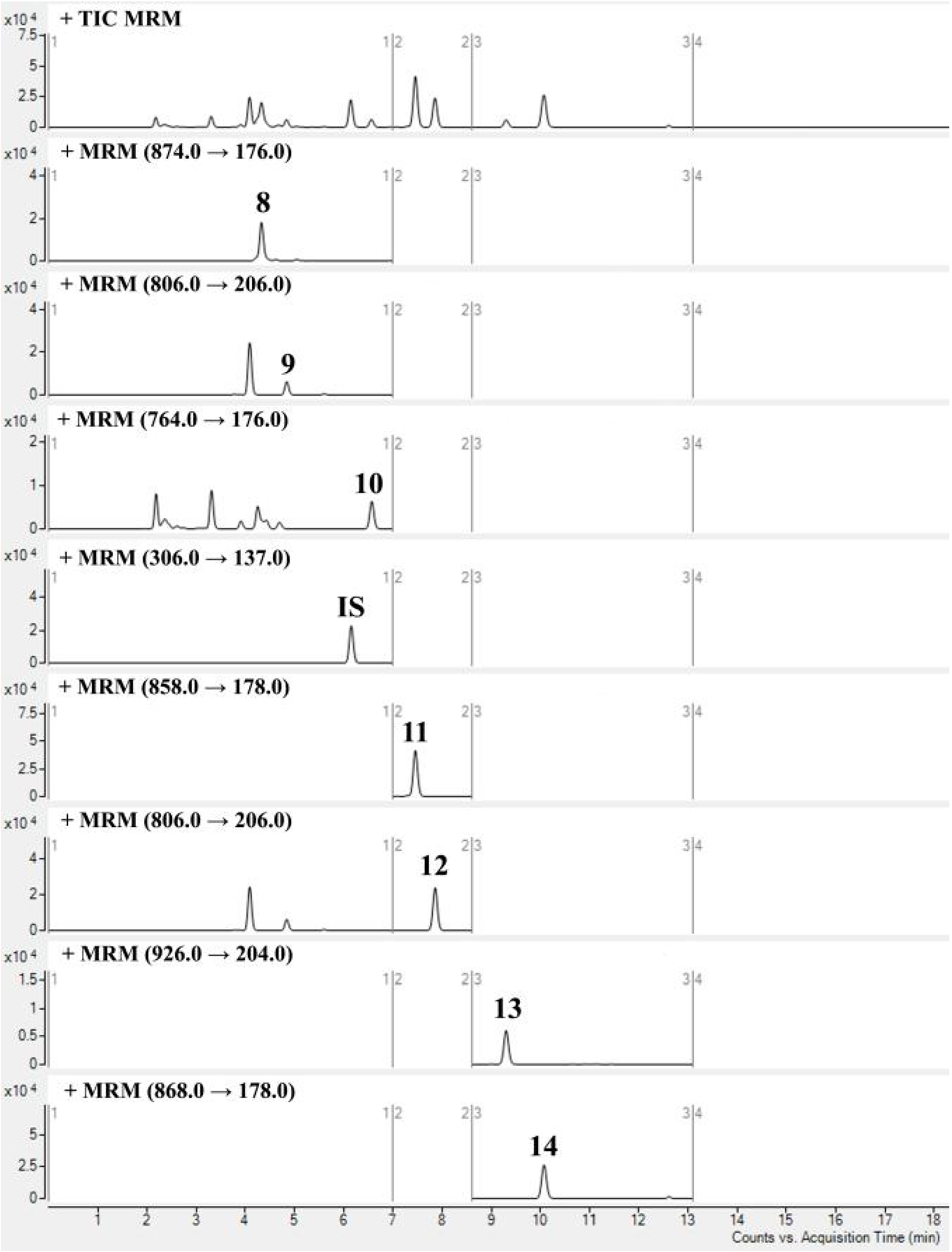


**Figure S3. Representative MRM chromatograms for investigated alkanoids in TGTs (S2) using acetonitrile-water as mobile phase**

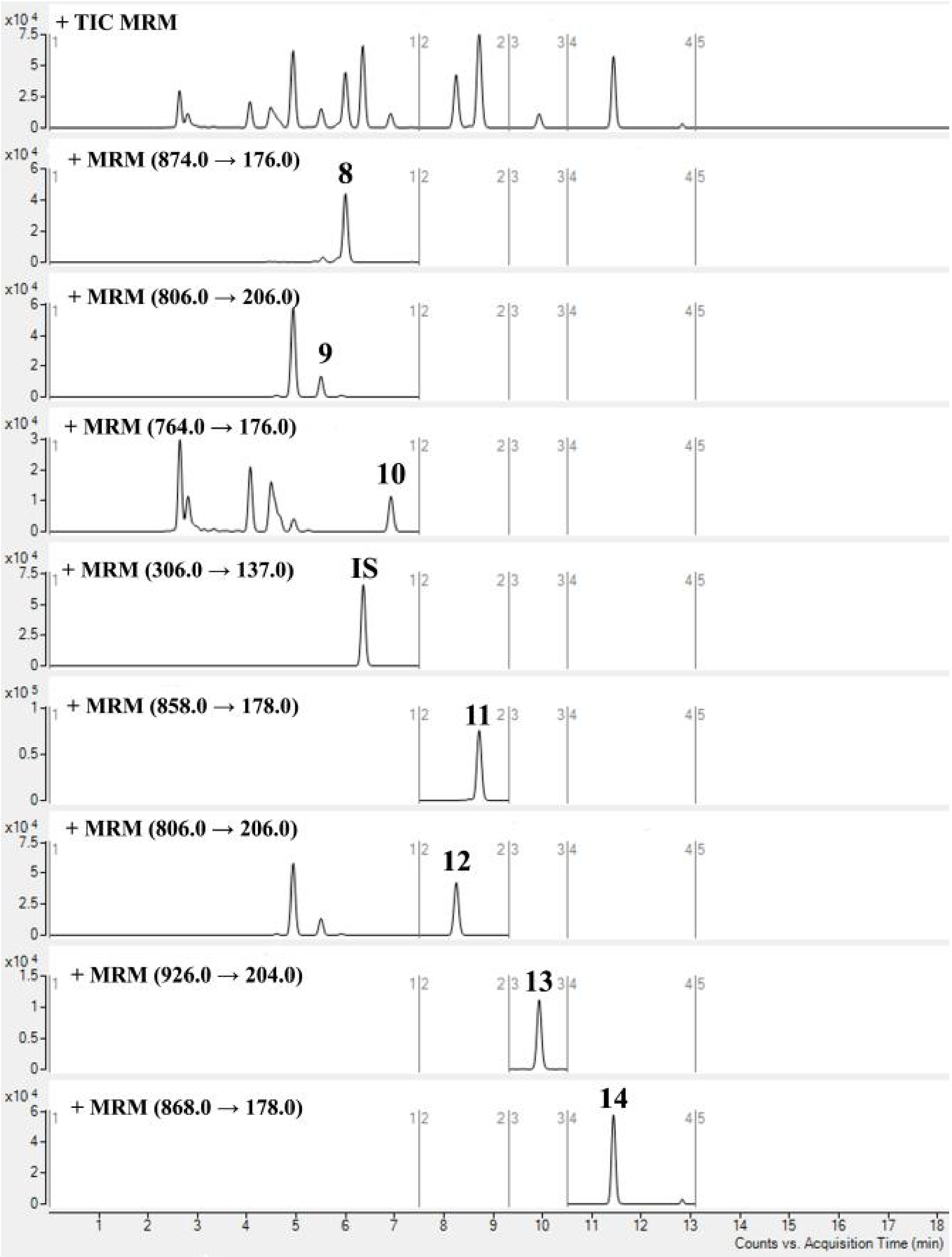


**Figure S4. Representative MRM chromatograms for investigated alkanoids in TGTs (S2) using acetonitrile-0.1% formic acid aqueous solution as mobile phase**

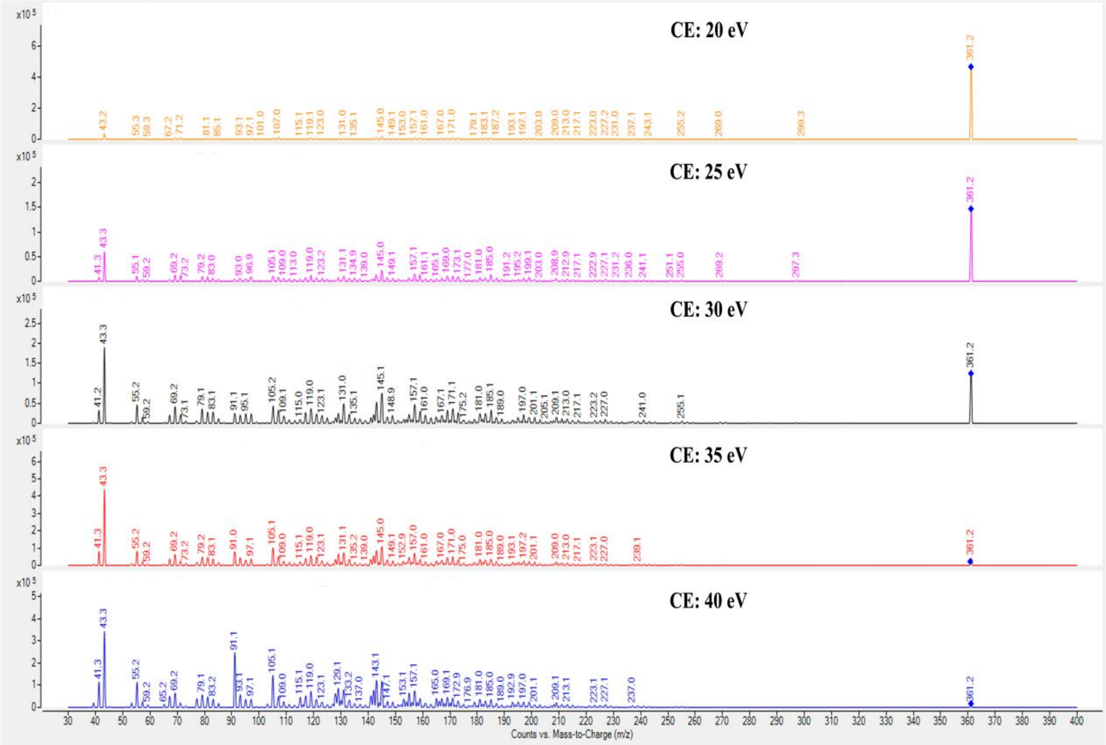


**Figure S5. The fragment profiles of triptolide with different collision energy**

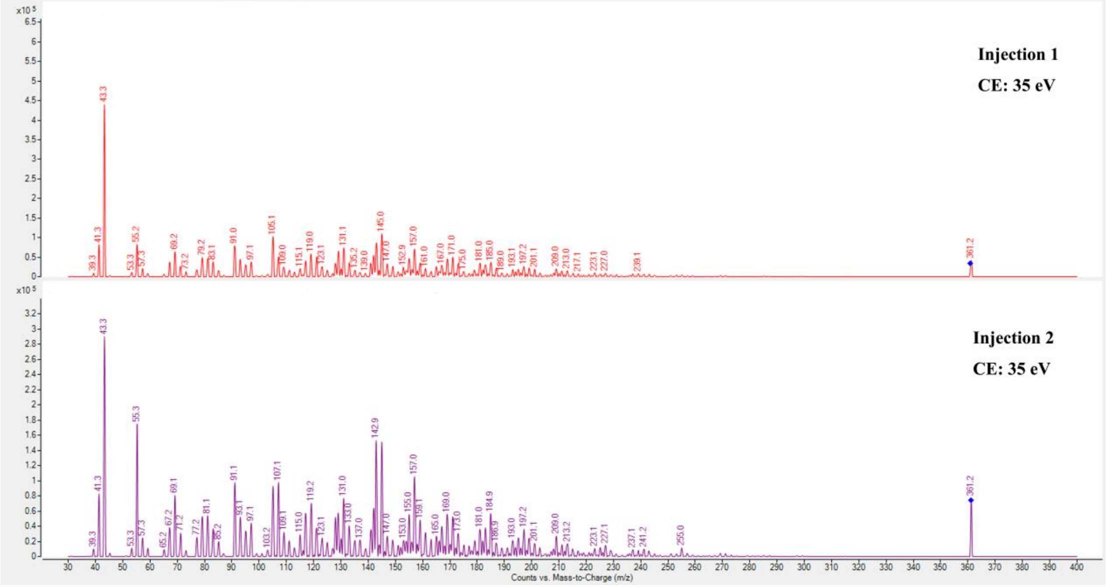


**Figure S6. The fragment profiles of triptolide with 35 eV of collision energy in two injections**

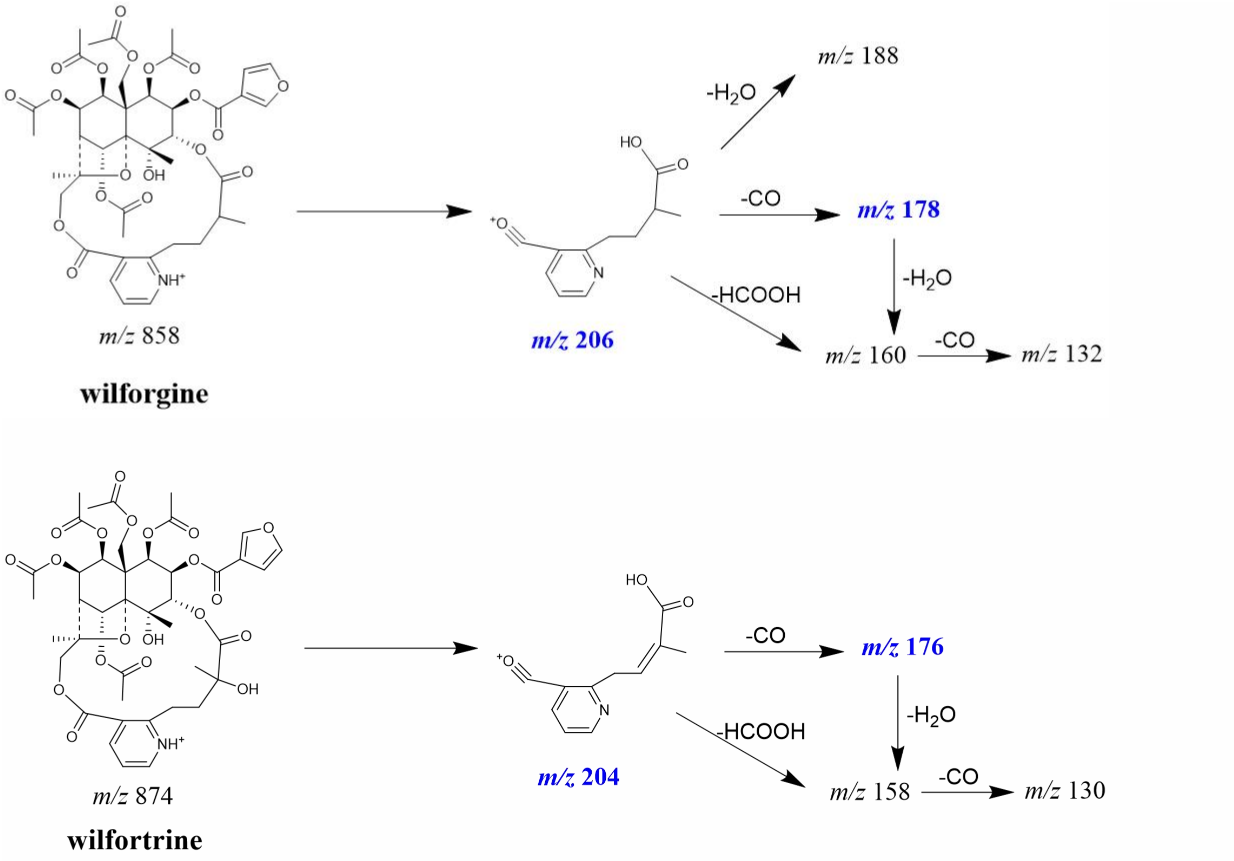


**Figure S7. The main fragmentation patterns of the alkaloids**

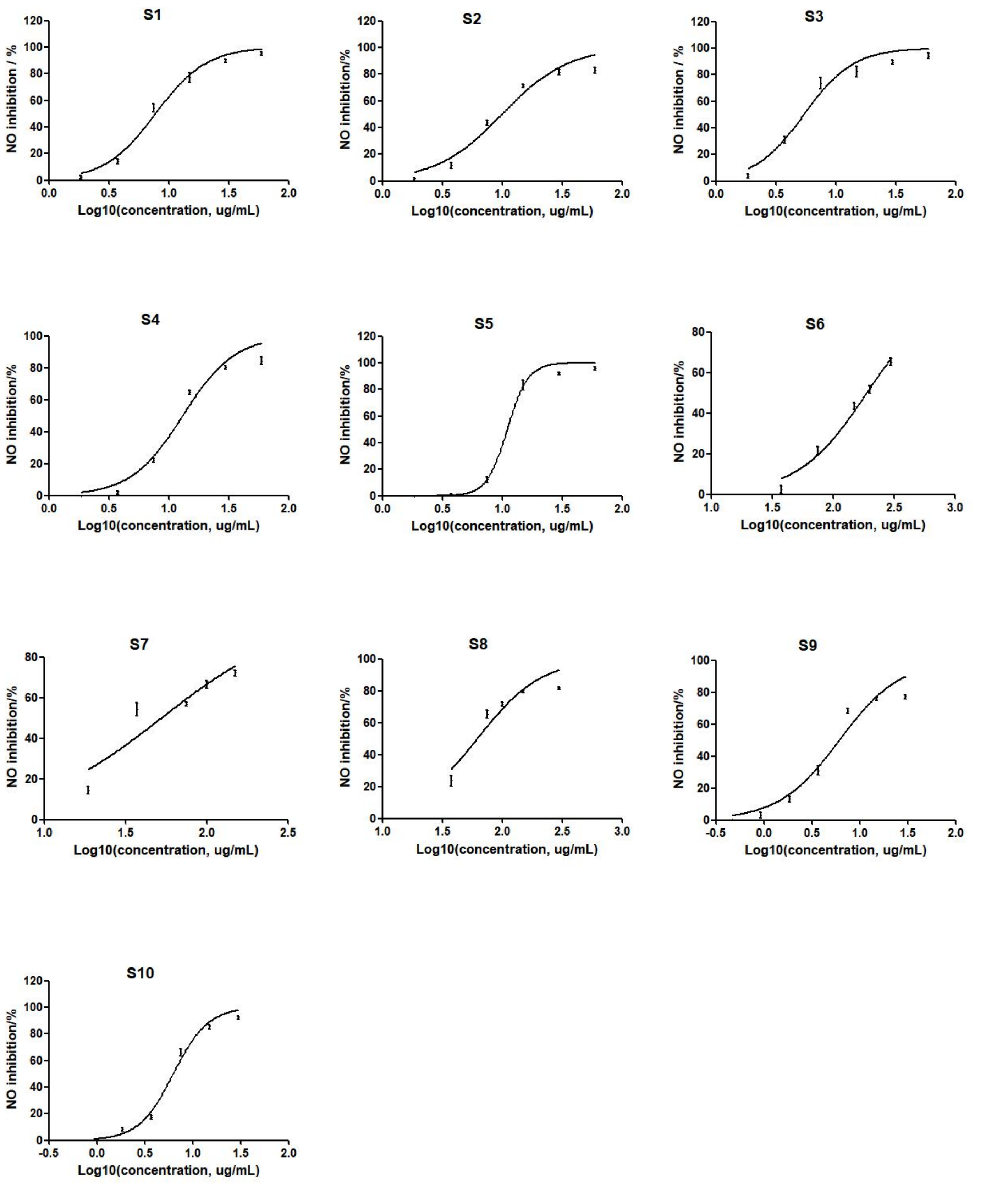


**Figure S8. The NO inhibition curves of 10 batches of *Tripterygium* glycosides tablets (TGTs, S1–S10)**

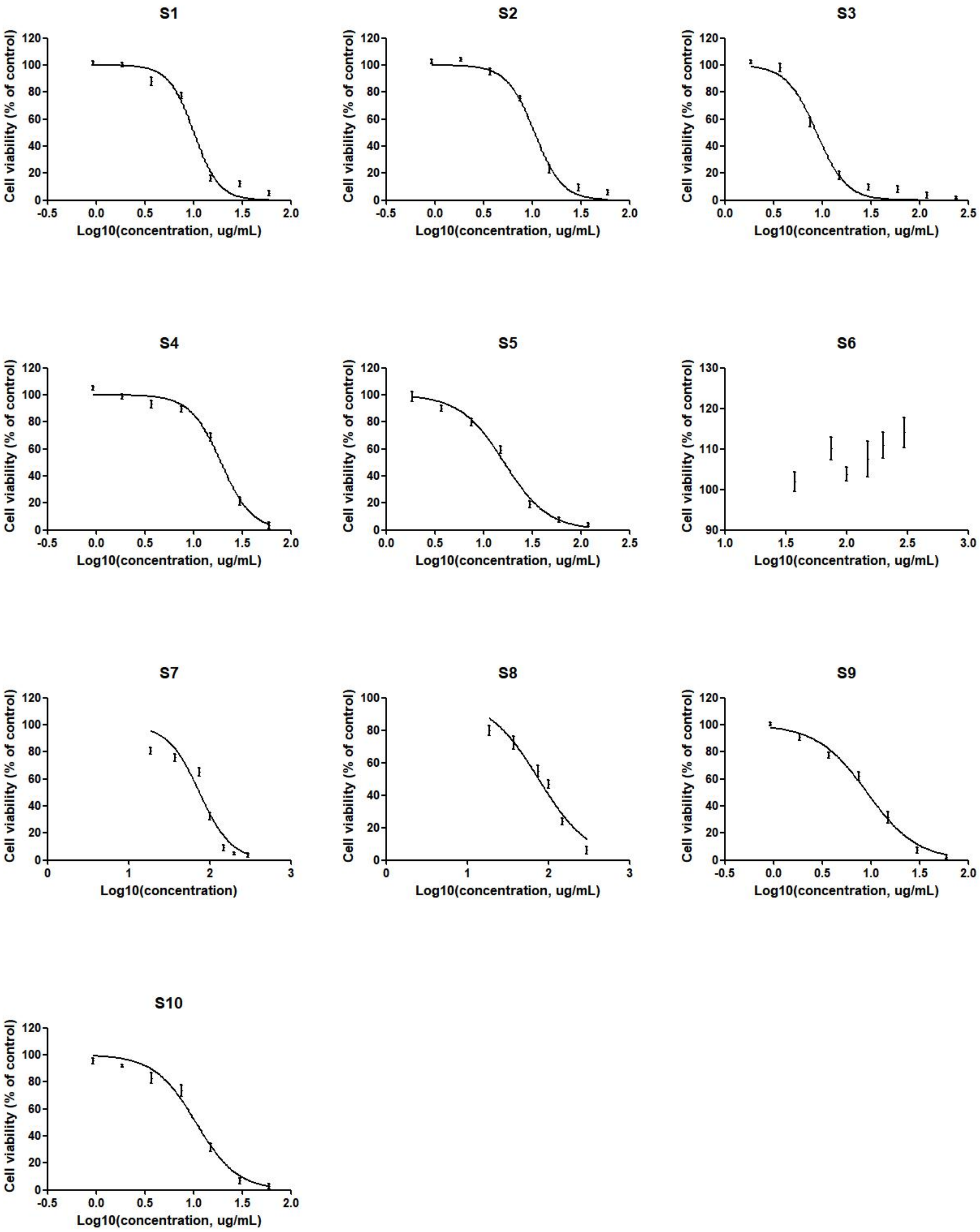


**Figure S9. The cytotoxicity curves against RAW 264.7 cells of 10 batches of TGTs (S1–S10)**

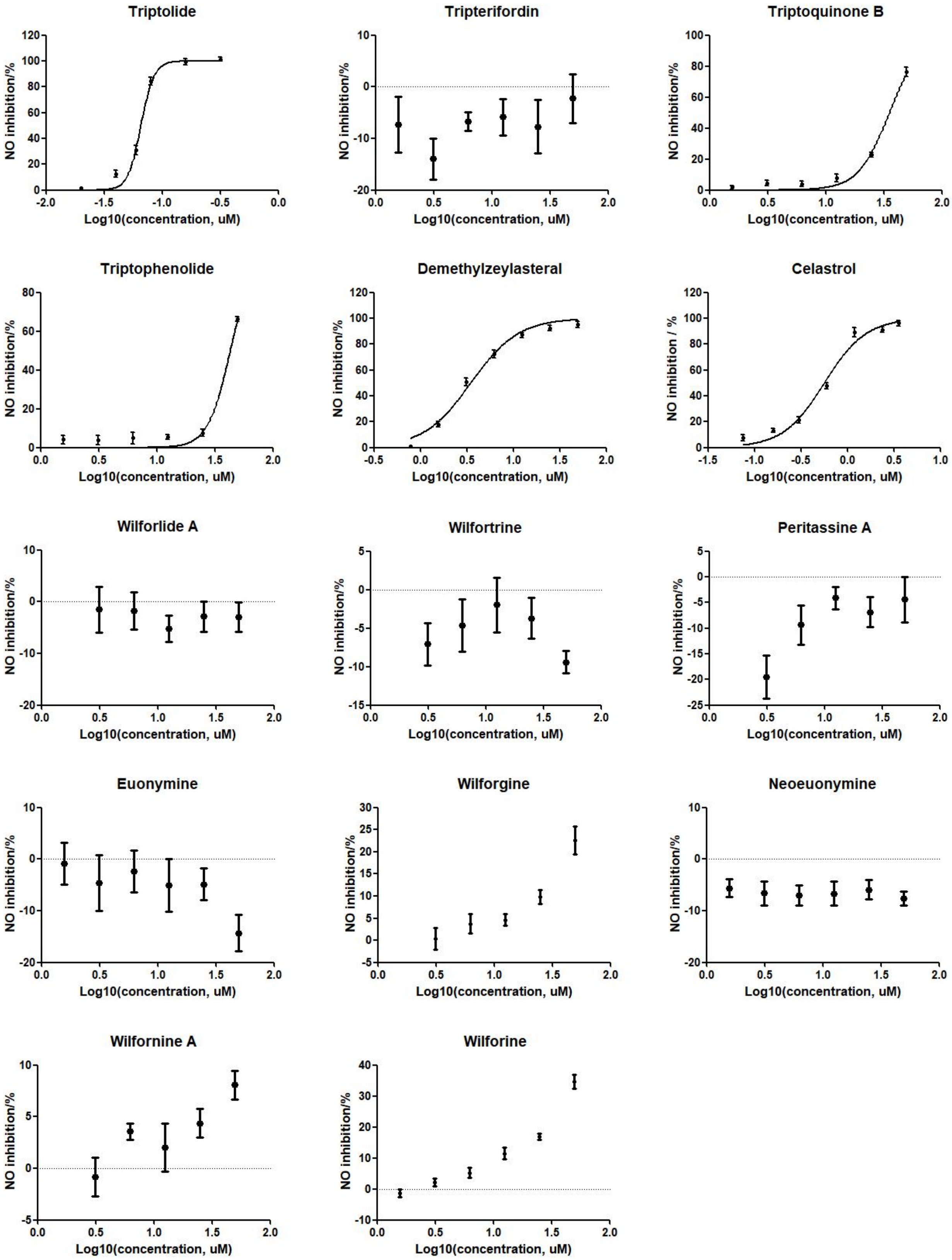


**Figure S10. The NO inhibition curves of 14 target compounds**

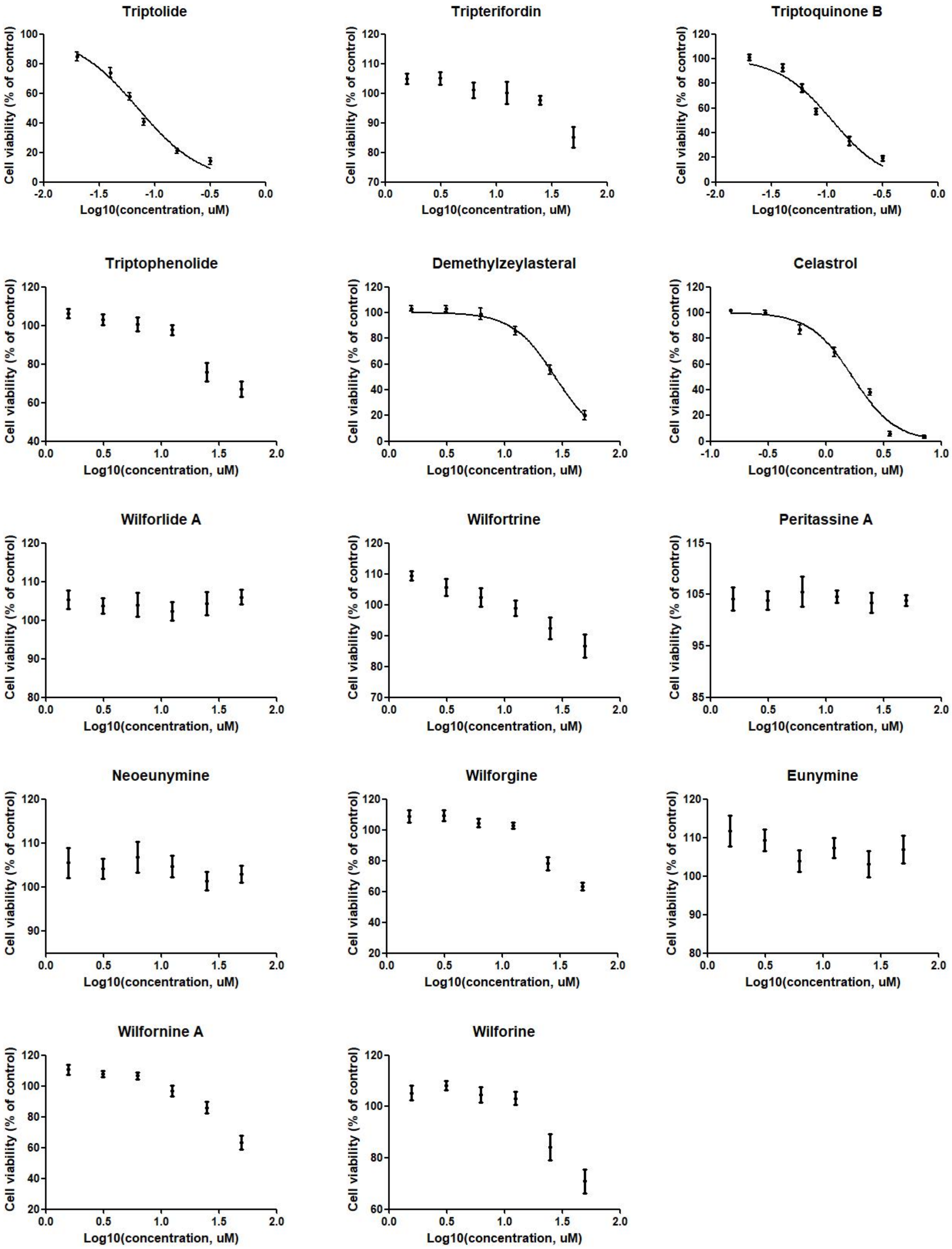


**Figure S11. The cytotoxicity curves against RAW 264.7 cells of 14 target compounds**

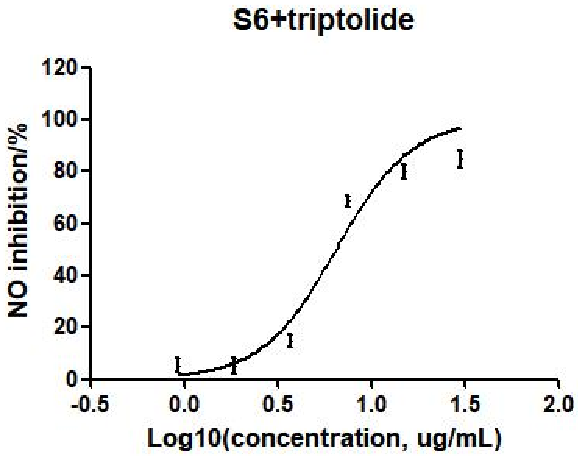


**Figure S12. The NO inhibition curve of S6 added with triptolide (10 μg/tablet)**

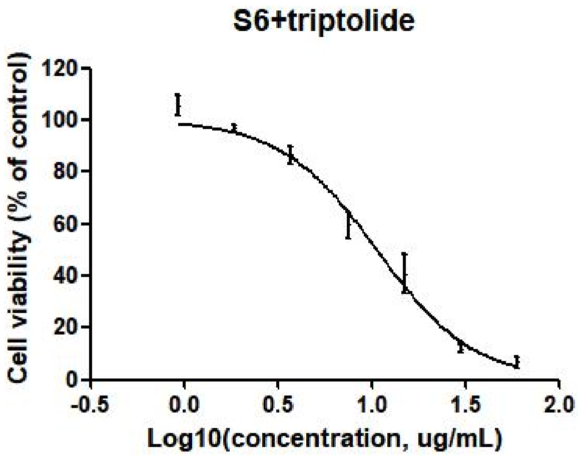


**Figure S13. The cytotoxicity curve against RAW 264.7 cells of S6 added with triptolide (10 μg/tablet)**

Table S1. Predicted absorption and metabolism parameters for investigated compounds by SwissADME tool

| Compound | GI absorption | BBB permeant | P-gp substrate | CYP1A2 inhibitor | CYP2C19 inhibitor | CYP2C9 inhibitor | CYP2D6 inhibitor | CYP3A4 inhibitor | Log Kp (skin permeation, cm/s) | Bioavailability Score |
|---|---|---|---|---|---|---|---|---|---|---|
| triptolide | High | No | Yes | No | No | No | No | No | -8.34 | 0.55 |
| tripterifordin | High | Yes | Yes | No | No | No | No | No | -5.52 | 0.55 |
| triptoquinone B | High | Yes | Yes | No | No | No | No | No | -6.41 | 0.55 |
| triptophenolide | High | Yes | Yes | No | Yes | Yes | No | Yes | -5.33 | 0.55 |
| demethylzeylasteral | Low | No | Yes | No | No | Yes | No | Yes | -4.82 | 0.56 |
| celastrol | Low | No | Yes | No | No | Yes | No | Yes | -4.83 | 0.85 |
| wilforlide A | High | No | No | No | No | Yes | No | No | -4.15 | 0.55 |
| wilfortrine | Low | No | Yes | No | No | No | No | No | -11.42 | 0.17 |
| peritassine A | Low | No | Yes | No | No | No | Yes | No | -10.90 | 0.17 |
| neoeunymine | Low | No | Yes | No | No | No | Yes | No | -11.02 | 0.17 |
| wilforgine | Low | No | Yes | No | No | No | No | No | -10.65 | 0.17 |
| euonymine | Low | No | Yes | No | No | No | Yes | No | -10.87 | 0.17 |
| wilfornine A | Low | No | Yes | No | No | No | No | No | -10.66 | 0.17 |
| wilforine | Low | No | Yes | No | No | No | No | No | -1005 | 0.17 |
